# Supplementary material for: Impact of palladium/palladium hydride conversion on electrochemical CO2 reduction via in-situ transmission electron microscopy and diffraction
Source: Nat Commun. 2024 Jan 31;15:938. doi: 10.1038/s41467-024-45096-3 (PMC10831057; doi:10.1038/s41467-024-45096-3)
Supplement: Supplementary file 1 — Supplementary Information [file 41467_2024_45096_MOESM1_ESM.pdf]

Supplementary Information

Impact of Palladium/Palladium Hydride Conversion on Electrochemical CO_2 Reduction via In-Situ Transmission Electron Microscopy and Diffraction

Ahmed M. Abdellah[1], Fatma Ismail[1], Oliver W. Siig[2], Jie Yang,[3] Carmen M. Andrei[4], Liza-Anastasia DiCecco[5], Amirhossein Rakhsha[1], Kholoud E. Salem[1], Kathryn Grandfield[3,5], Nabil Bassim[3,4], Robert Black[6], Georg Kastlunger[7*], Leyla Soleymani[5,7], Drew Higgins[1,4*]

1- Department of Chemical Engineering, McMaster University, Hamilton, Ontario L8S 4L7, Canada
2- CatTheory, Physics Department, Denmark Technical University, Kongens Lyngby 2800, Denmark
3- Department of Materials Science and Engineering, McMaster University, Hamilton, ON, Canada
4- Canadian Centre for Electron Microscopy, McMaster University, Hamilton, Canada
5- School of Biomedical Engineering, McMaster University, Hamilton, Canada
6- National Research Council of Canada, Energy, Mining, and Environment Research Centre, 2620 Speakman Drive, Mississauga, ON L5K 2L1, Canada
7- Department of Engineering Physics, McMaster University, Hamilton, Canada

*higgid2@mcmaster.ca

*geokast@dtu.dk

Electrochemical configurations used in this work:

1- **In-situ liquid phase TEM Protochips Reactor**

The in-situ TEM setup illustrates the whole configuration utilized in performing in-situ liquid phase TEM measurements. Supplementary Fig. 1a reveals the three-electrode configuration of the in-situ TEM tip. Supplementary Fig. 1b shows the different components of the system including a potentiostat, syringe pump, and the in-situ TEM reactor.

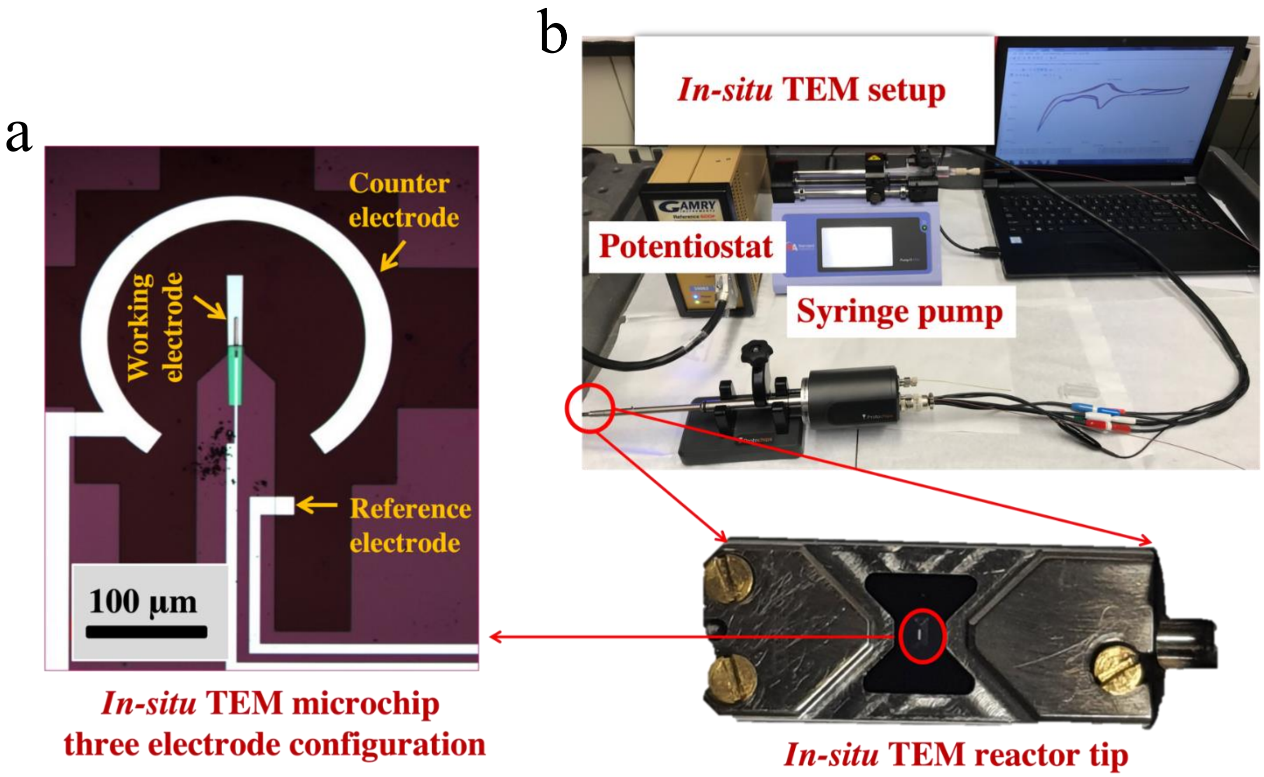

Supplementary Fig. 1 Protochips setup of the in-situ TEM liquid cell: **a** Three-electrode system configuration of the E-chip under light microscope. **b** Protochip system components comprising potentiostat, syringe pump, and TEM holder.

Supplementary note 1:

Details of the Beam dose calculations

$$\text{Beam Dose} = \frac{\text{Beam Current} \times \text{Frame time}}{\text{View Area}}$$

$$\text{View Area} = \text{Pixel number} \times \text{Pixel size}$$

$$\text{Frame time} = \text{Dewll time} \times \text{Pixel number}$$

2- Large-format electrode reactor:

Benchtop measurements were conducted to evaluate the CO_2R activity/selectivity of Pd electro-deposited on a glassy carbon electrode, Pt foil was used as a counter electrode and Ag/AgCl as a reference electrode. All subsequent potentials are given against the reversible hydrogen electrode (RHE) based on equation 1 and the calibration of Pt as shown in Figure S2.

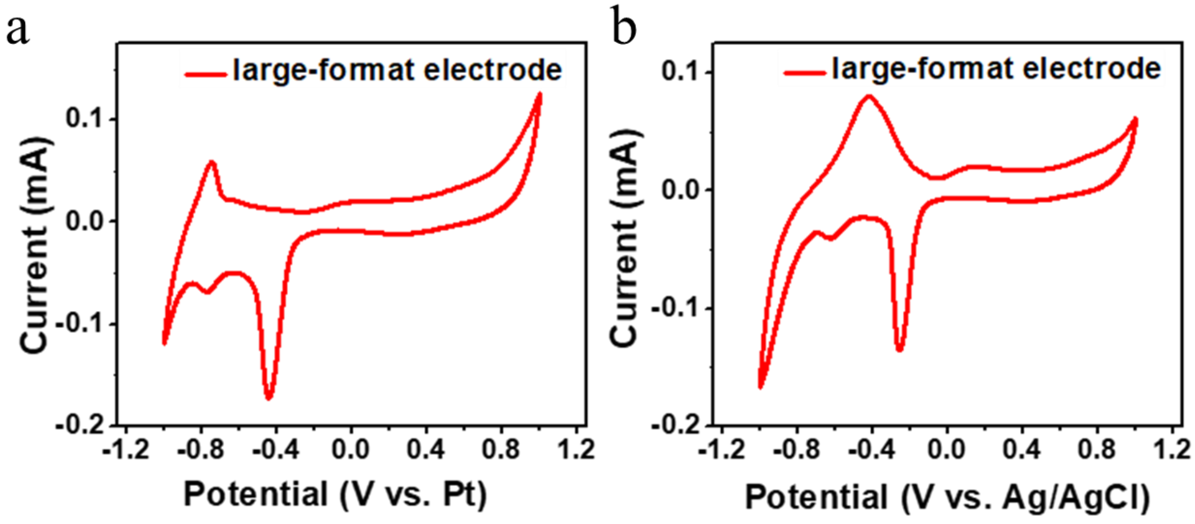

Supplementary Fig. 2 Cyclic voltammetry features of Pd electrodeposited on large-format glassy carbon electrode using **a** Pt as a reference electrode and **b** Ag/AgCl as a reference electrode, both in N_2-saturated 0.1M $KHCO_3$ at a scan rate of 100mV/sec. where E vs RHE= E vs Pt + 0.76V.

Supplementary Note 2:

In this study, the floating potentiostat's potential against the reversible hydrogen electrode (RHE)[1] is estimated using the following Eq:

$$E_{RHE} = E_{Ag/Agcl} + 0.197V + 0.059 \times pH + R \times I$$

Where;

R: Ohmic resistance between working and reference electrode / Ω

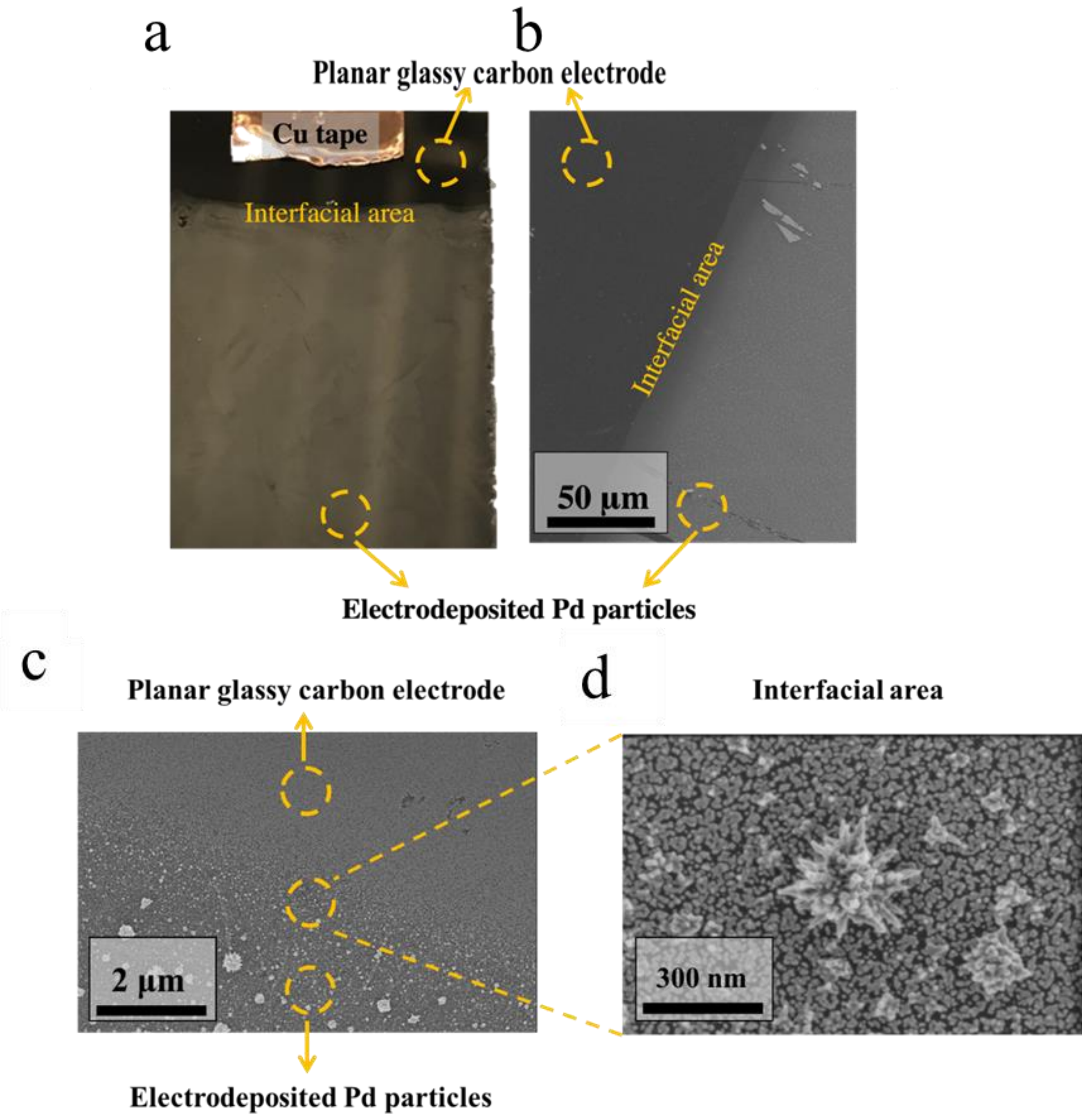

Supplementary Fig. 3 **a** Optical micrograph of Pd electrodeposited on the large-format glassy carbon electrode. **b-d** SEM images of the electrodeposited Pd particles at different magnifications.

Calculation of liquid thickness for in-situ LP-TEM using EELS:

The relative liquid thickness (t) in terms of the mean free path of the electrons (λ) can be estimated from the plasmon scattering peak in the EELS spectra by using the Beer–Lambert's law equation[2].

$$\frac{t}{\lambda} = -\ln\left(\frac{I^{\circ}}{I_t}\right)$$

Where;

I°: the number of unscattered electrons in the zero-loss peak

It: the total number of incident electrons

From this equation, the relative thickness of the liquid (tliquid/λ liquid) can be estimated by subtracting the relative thickness of the two dry 50 nm SiNx membranes $(t/\lambda)SiN_x$) from the total relative thickness of the entire liquid-filled electrochemical cell ($(t/\lambda)SiNx\text{-}liquid\text{-}SiN_x$) as shown in the following equation:

$$\frac{t_{liquid}}{\lambda_{liquid}} = \left(\frac{t}{\lambda}\right)_{SiN_x-liquid-SiN_x} - 2\left(\frac{t}{\lambda}\right)_{SiN_x}$$

$(t/\lambda)_{SiNx}$ was calculated to be 0.38 and $(t/\lambda)_{SiNx\text{-}liquid\text{-}SiNx}$ was calculated to be 1.23, leading to a calculated $t_{liquid}/\lambda_{\ liquid}$ value of 0.47. Assuming the inelastic mean free path of water to be 106 nm [3], the liquid thickness within the LP-TEM electrochemical cell was calculated to be ca. 50nm.

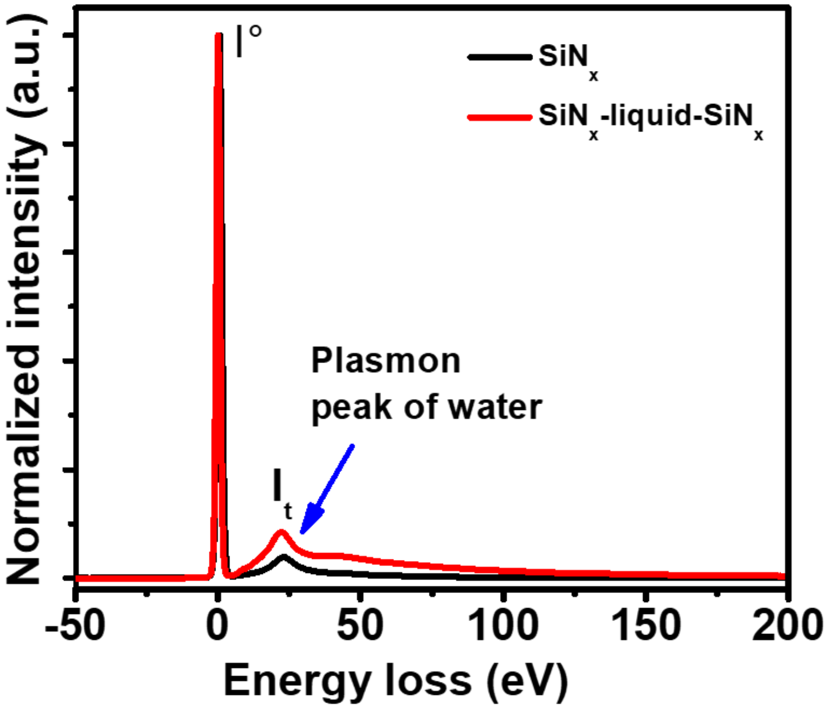

Supplementary Fig. 4 Illustrates STEM-EELS measurements of Zero-loss peak for a dry 50 nm SiN_x window compared to thin film liquid filled LP-TEM electrochemical cell (SiN_x-liquid-SiN_x) at an applied potential of -0.2 V vs. RHE in CO_2 saturated 0.1M $KHCO_3$.

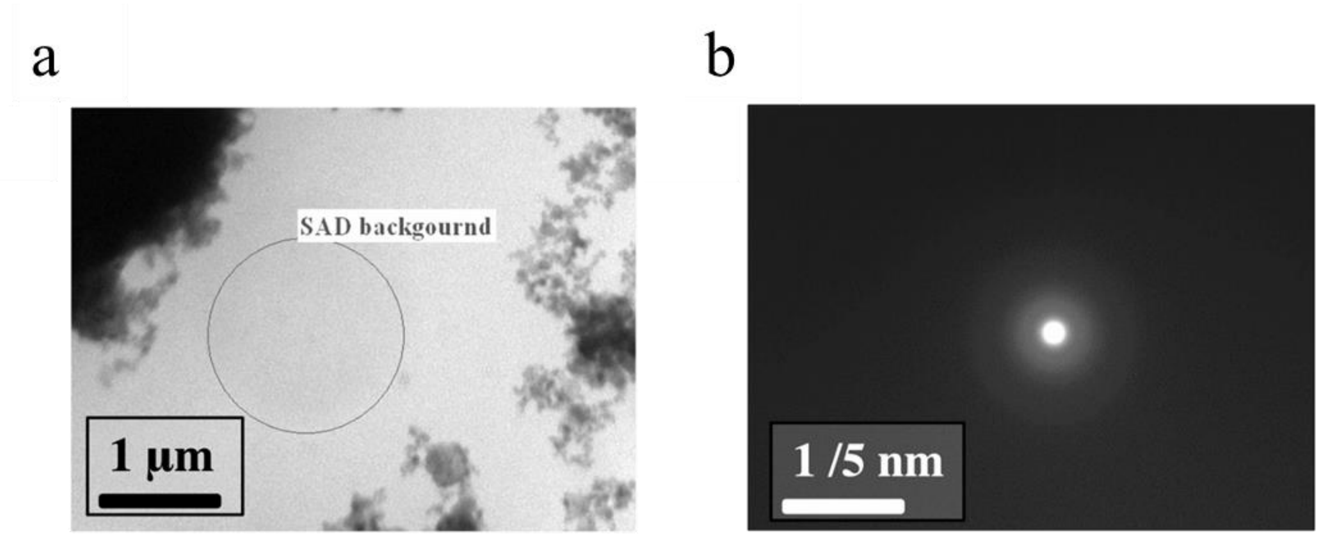

Supplementary Fig. 5 a In-situ LP-TEM imaging indicating the region where background select area diffraction (SAD) measurements were conducted. **b** SAD pattern of the background from the in-situ LP-TEM reactor in CO_2 saturated 0.1M $KHCO_3$.

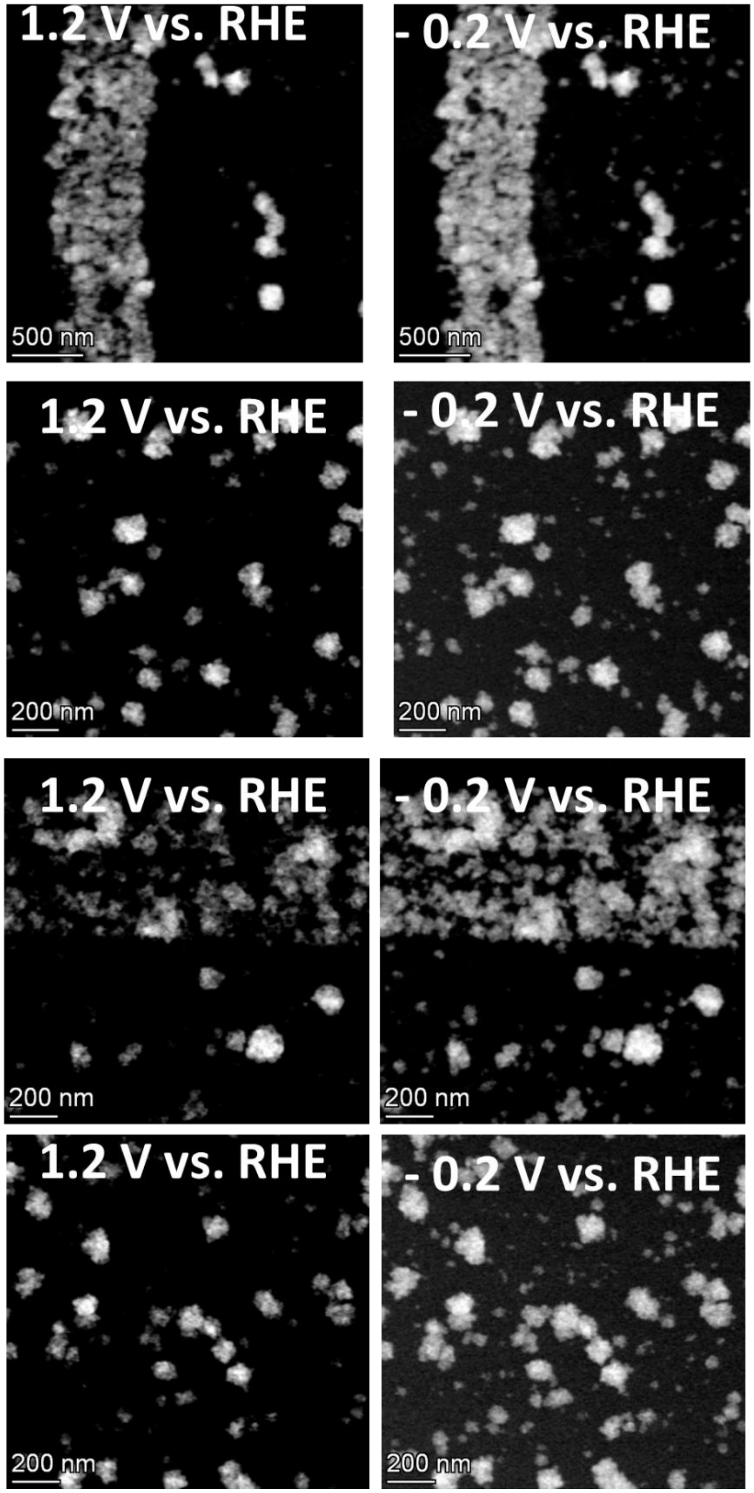

Supplementary Fig. 6 HAADF-STEM images of Pd particles held at an electrode potential ranging between 1.2 V and -0.2 V vs RHE in CO_2 saturated 0.1M $KHCO_3$, demonstrating the enlargement of the Pd particles as a function of potential.

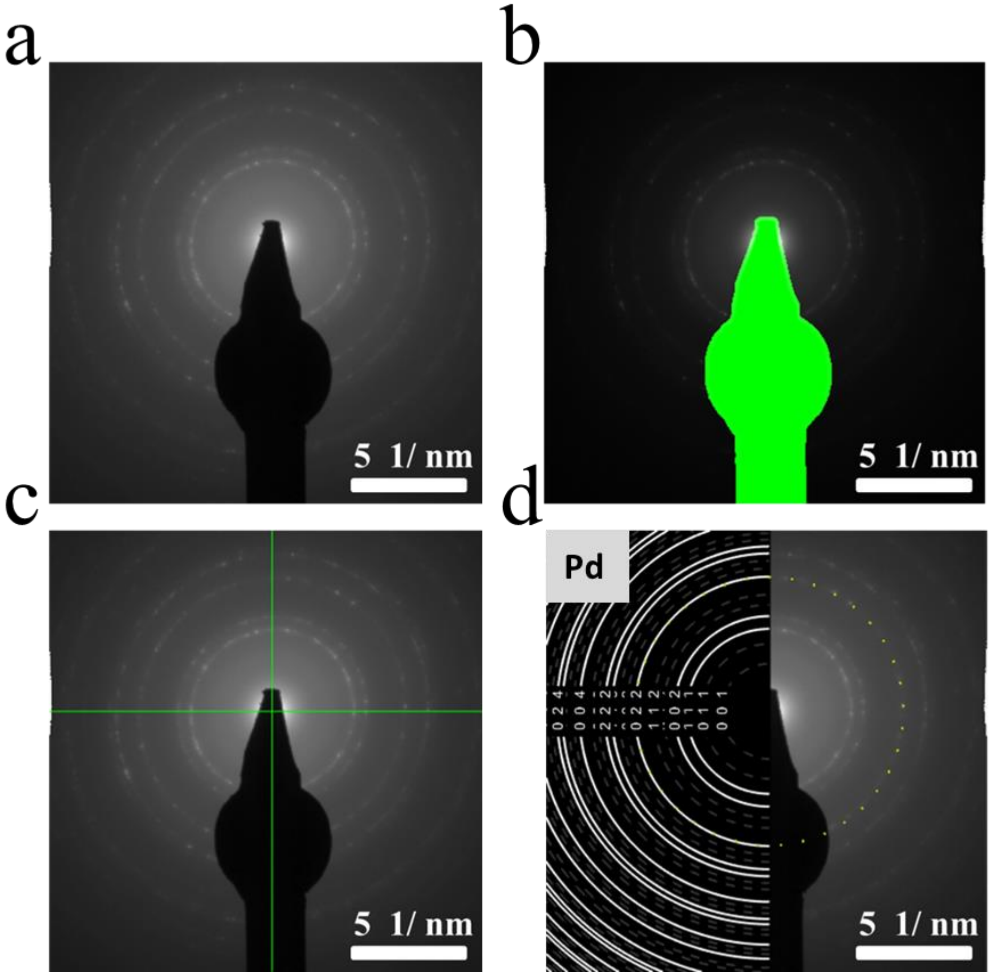

Supplementary Fig. 7 Illustration of CrysTBox ringGUI – analysis under an applied potential of 1.2 V vs RHE in CO_2 saturated 0.1M $KHCO_3$. **a** Original image of the SAD pattern. **b** Beam stopper detection. **c** Diffraction ring centering. **d** Individual lattice vectors are crystallographically identified and the zone axis is calculated.

<u>Supplementary note 3:</u>

The SAD analysis was performed using the software CrysTBox ringGUI 1.16 by Miloslav Klinger. A comparison of the theoretical and experimental values of the Pd particle analysis under an applied potential of 1.2 V vs RHE in CO_2 saturated 0.1M $KHCO_3$ is provided in Supplementary Table S1.

Supplementary Table1: Summary of SAD analysis for the metallic Pd under an applied potential of 1.2 V vs RHE.

Ring identification (Pd)				
Plane	Radius (1/nm)		d-spacing (nm)	
	theoretical	measured	theoretical	measured
(111)	4.45	4.44	0.22	0.23
(002)	5.14	5.05	0.19	0.20
(022)	7.27	7.24	0.14	0.14
(113)	8.53	8.50	0.12	0.12

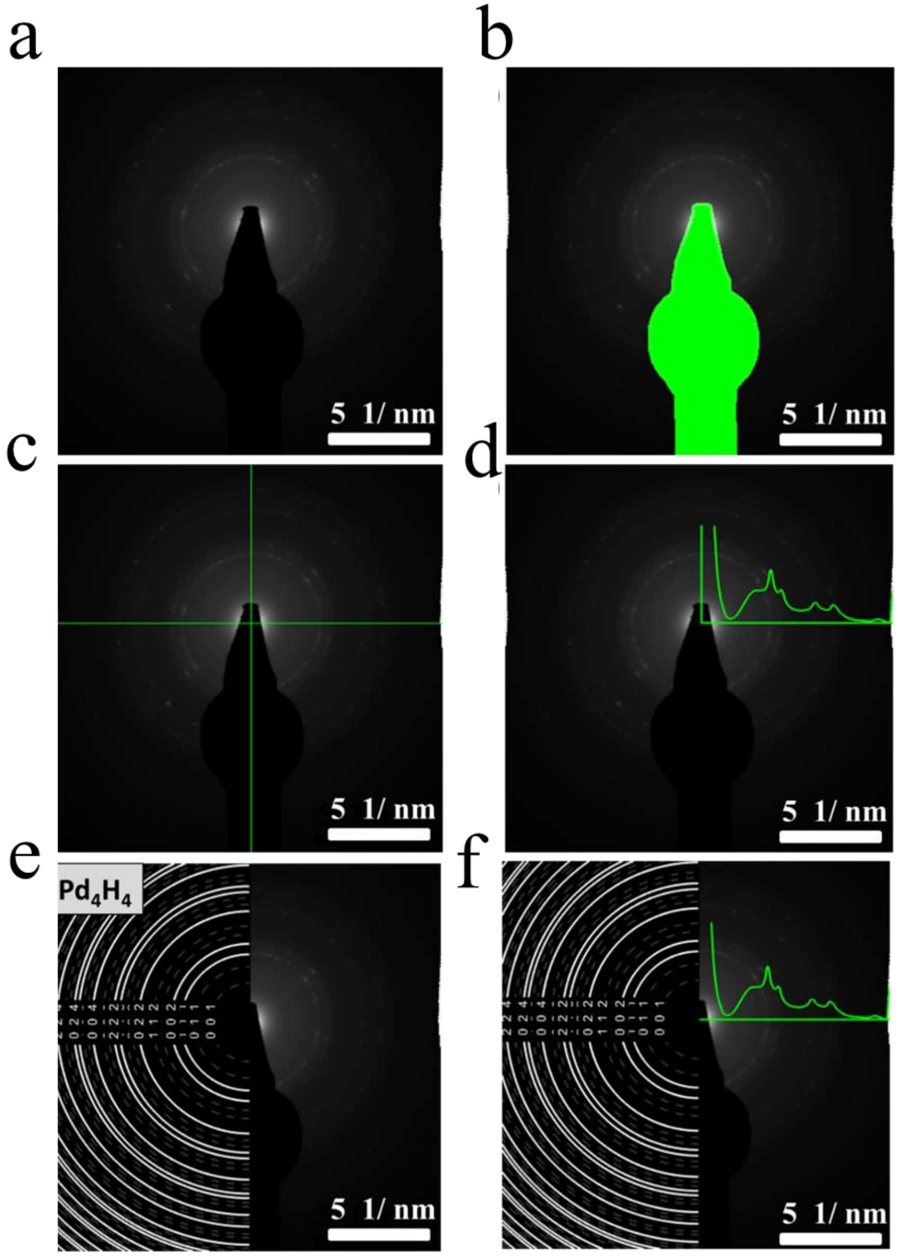

Supplementary Fig. 8 Illustration of CrysTBox ringGUI – analysis of PdH_x formed under an applied potential of -0.2 V vs. RHE in CO_2 saturated 0.1M $KHCO_3$. **a** original image. **b,** Beam stopper detection. **c** Rings center. **d** Diffractogram profile - circular average. **e and f**, Individual lattice vectors are crystallographically identified and the zone axis is calculated.

Supplementary note 4:

Supplementary Table 2: Summary of SAD analysis of PdH_x formed under an applied potential of -0.2 V vs. RHE in CO_2 saturated 0.1M $KHCO_3$**,** demonstrate the comparison of the theoretical and experimental values of PdH_x

Ring identification (PdH_x)				
Plane	Radius (1/nm)		d-spacing (nm)	
	theoretical	measured	theoretical	measured
(111)	4.19	4.26	0.24	0.24
(002)	4.83	4.91	0.21	0.20
(022)	6.83	7.05	0.15	0.14
(113)	8.01	8.08	0.12	0.12

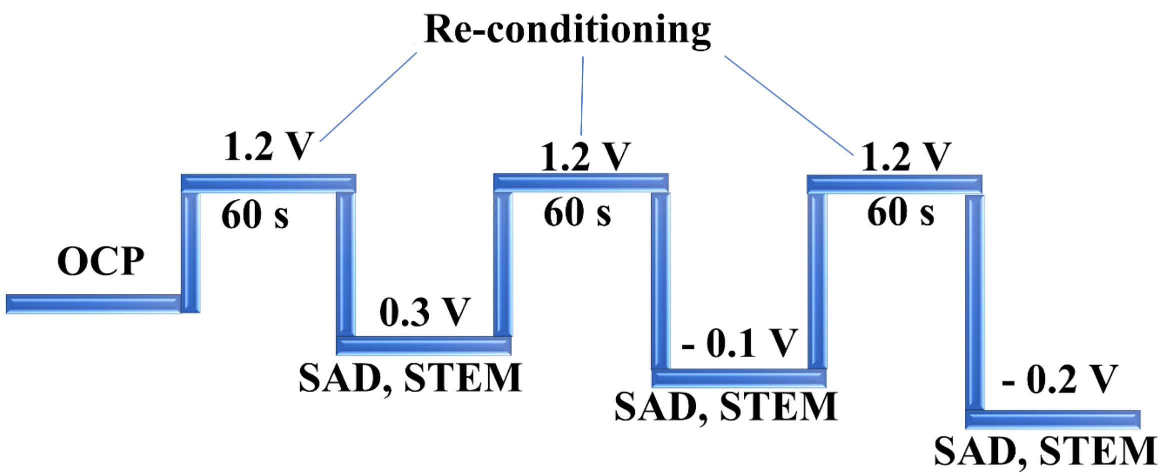

Supplementary Fig. 9 Illustration of the electrode potential profiles used during the in-situ SAD&STEM measurements (potentials given against the RHE).

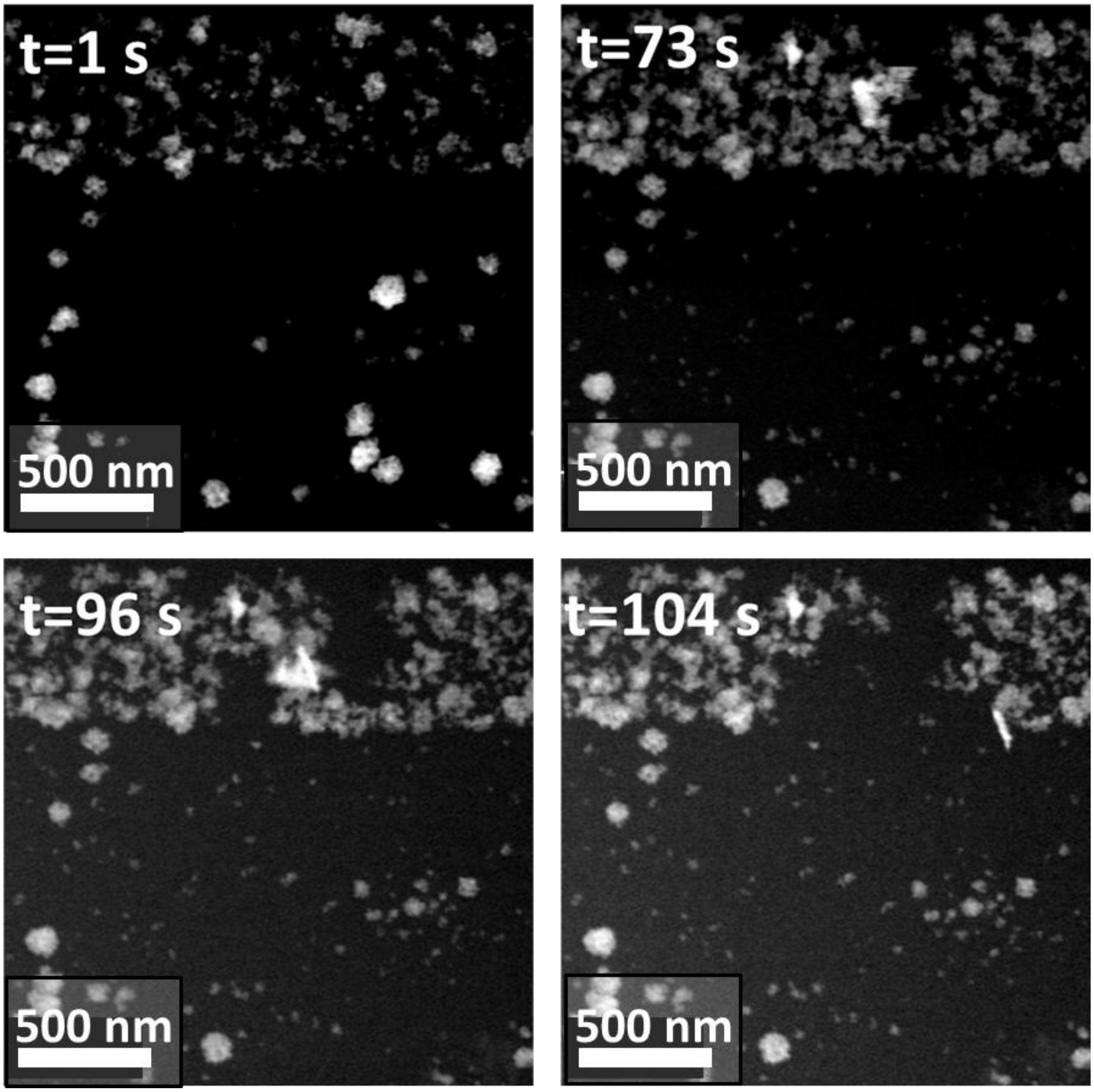

Supplementary Fig. 10 In-situ HAADF-STEM snapshots extracted from Supplementary Movie 4 illustrating Pd/PdH_x particle detachment from the glassy carbon working electrode under an applied potential of -0.2 V vs RHE in CO_2 saturated 0.1M $KHCO_3$ for varying time durations.

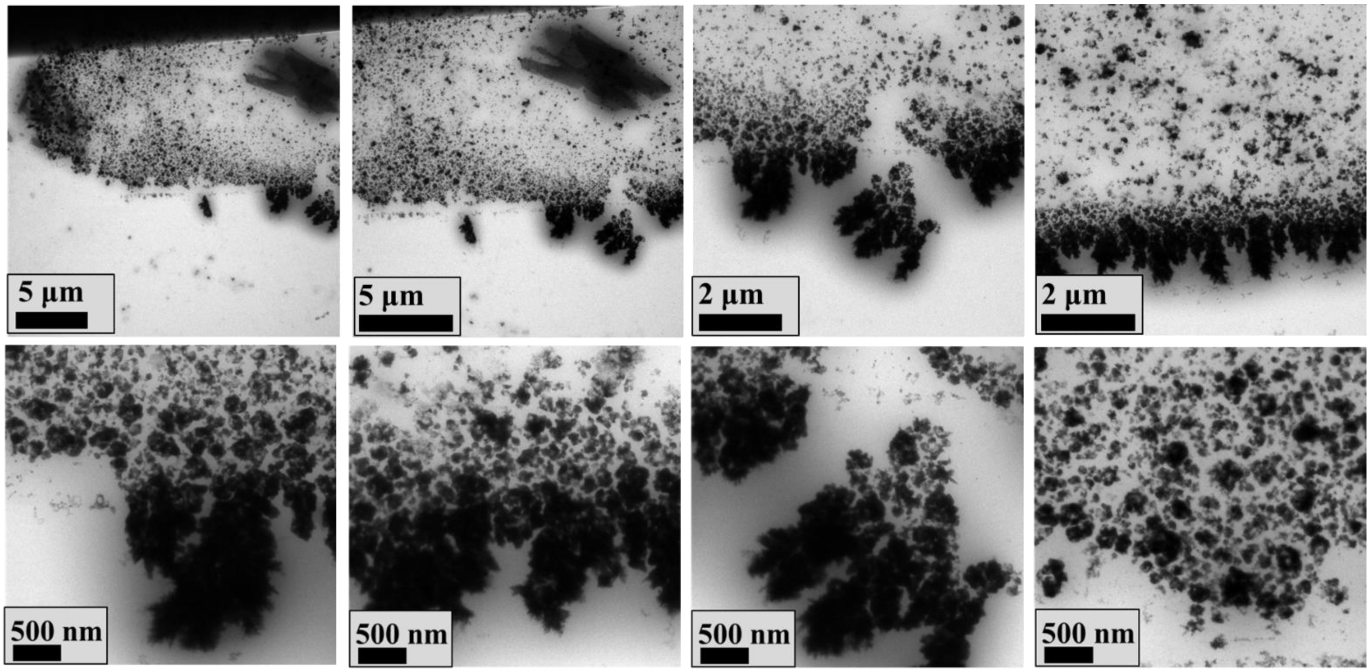

Supplementary Fig. 11 Ex situ TEM images of in-situ TEM working electrode after CO_2 electrolysis.

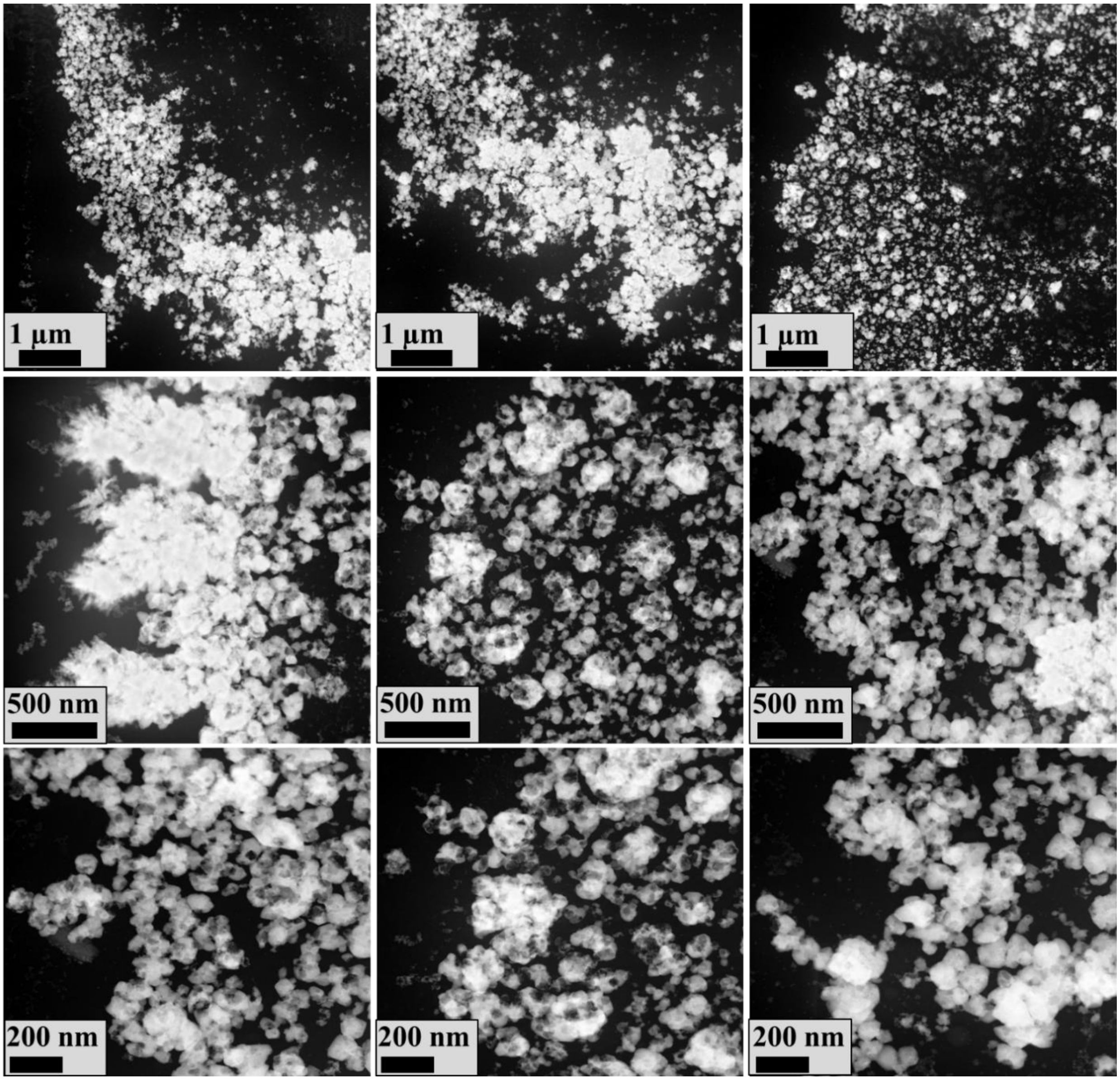

Supplementary Fig. 12 HAADF-STEM images of in-situ TEM working electrode after CO_2 electrolysis.

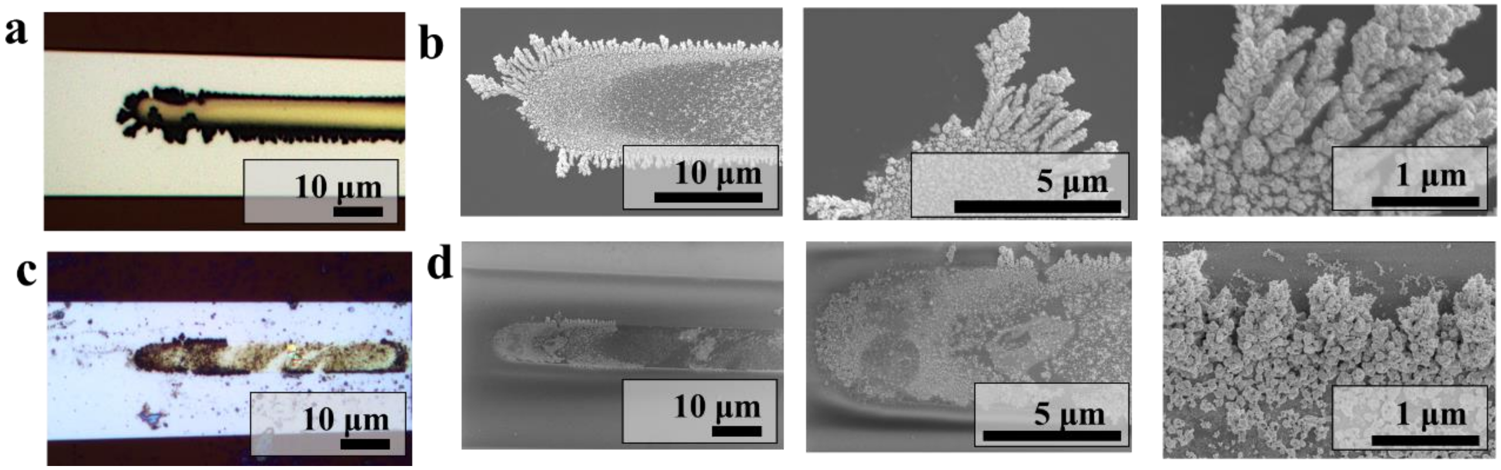

Supplementary Fig. 13 Optical (a-c) and SEM images (b-d) of Pd particle on the in-situ glassy carbon electrode: **a-b** before and **c-d** after CO_2 electrolysis.

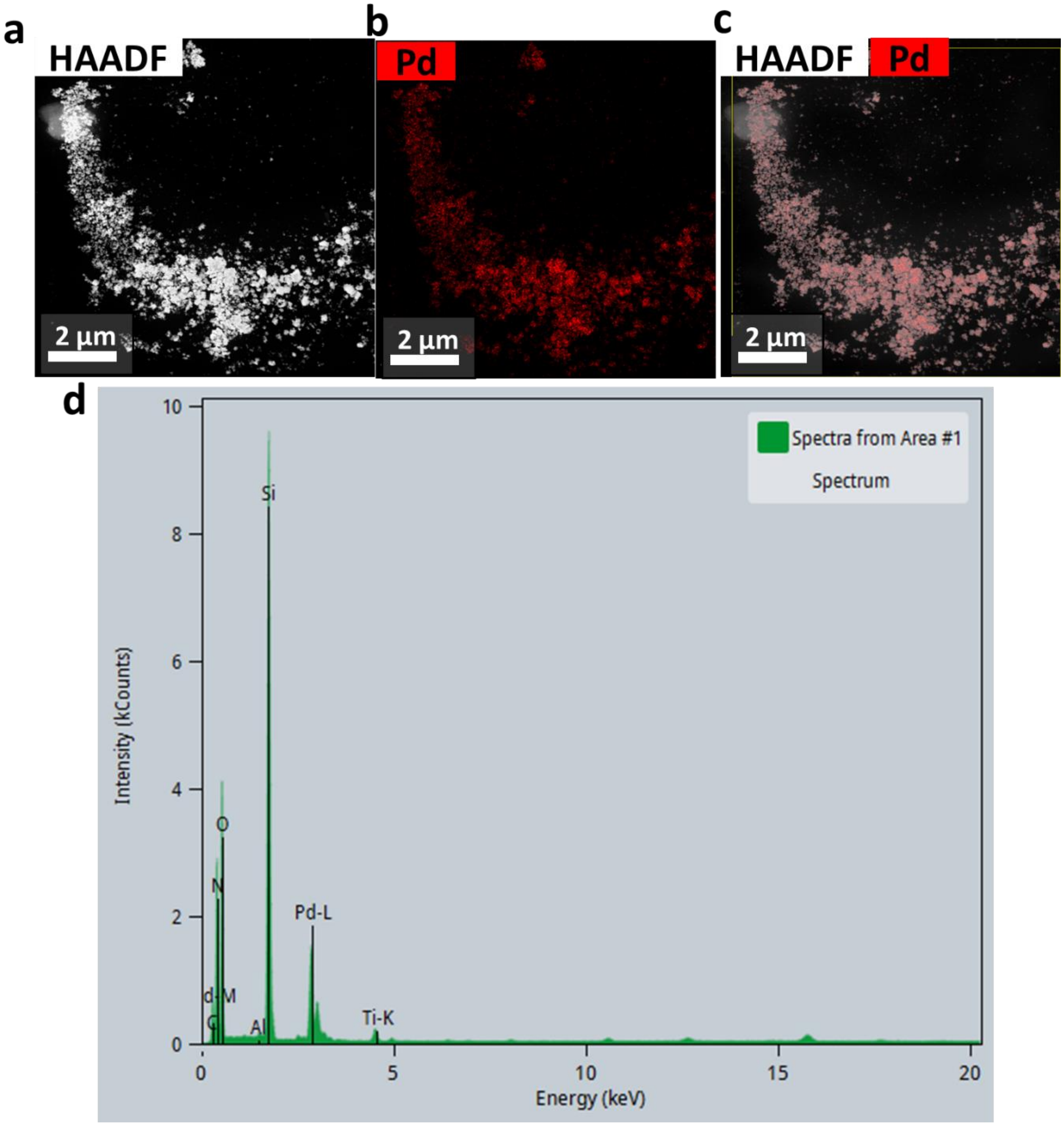

Supplementary Fig. 14 a HAADF-STEM, **b-c** HAADF-STEM-EDX mapping images and **d** EDX-spectrum of the Pd particle on the in-situ glassy carbon electrode after in-situ SAD measurements under CO_2 electrolysis conditions. Note that the source of Ti and Al peak in EDX spectrum in panel **d** comes from the lid of the in-situ TEM holder.

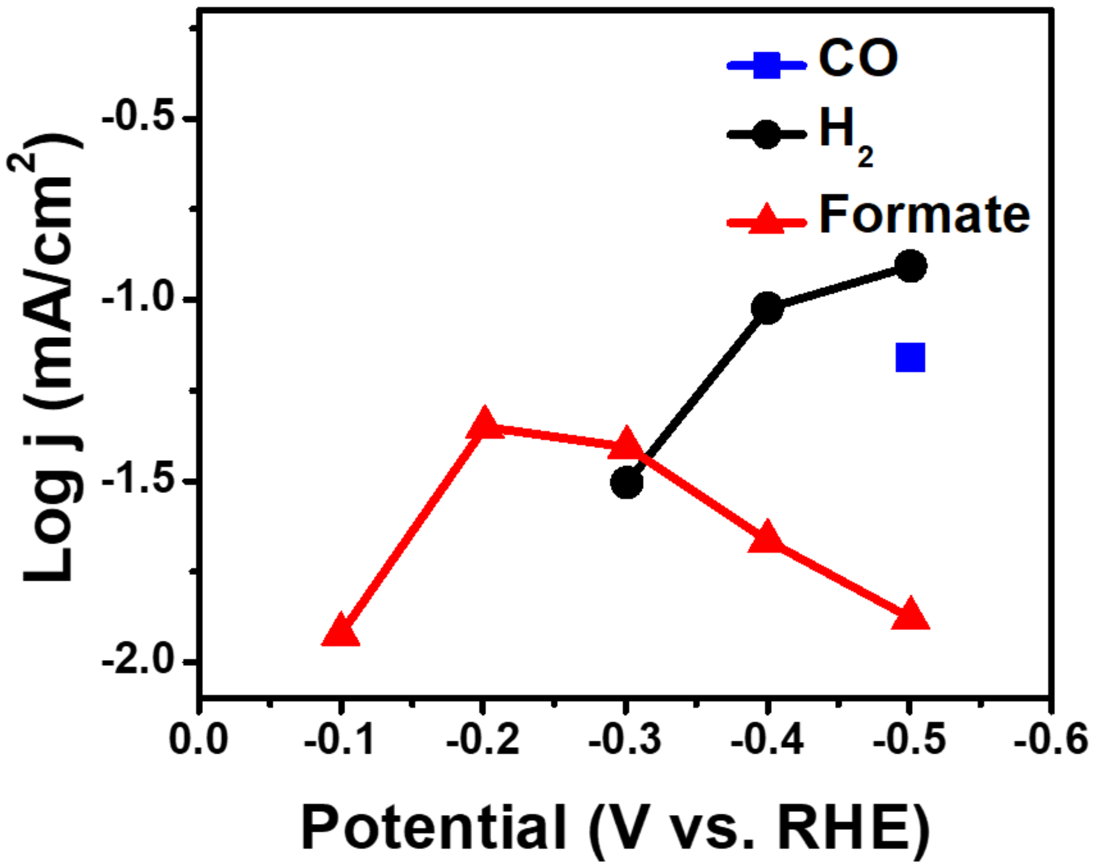

Supplementary Fig. 15 Tafel analysis for the CO_2R to CO, H_2 and formate in CO_2-saturated 0.1 M $KHCO_3$. Noted that the electrochemical CO_2 conversion test setup used in this work is associated with mass transport limitations; thus, the precise determination of Tafel plots is problematic. However, our total current densities are fairly low (< 0.5 mA/cm^2), which is in a range where mass transport limitations do not play a prominent role but cannot be neglected entirely.

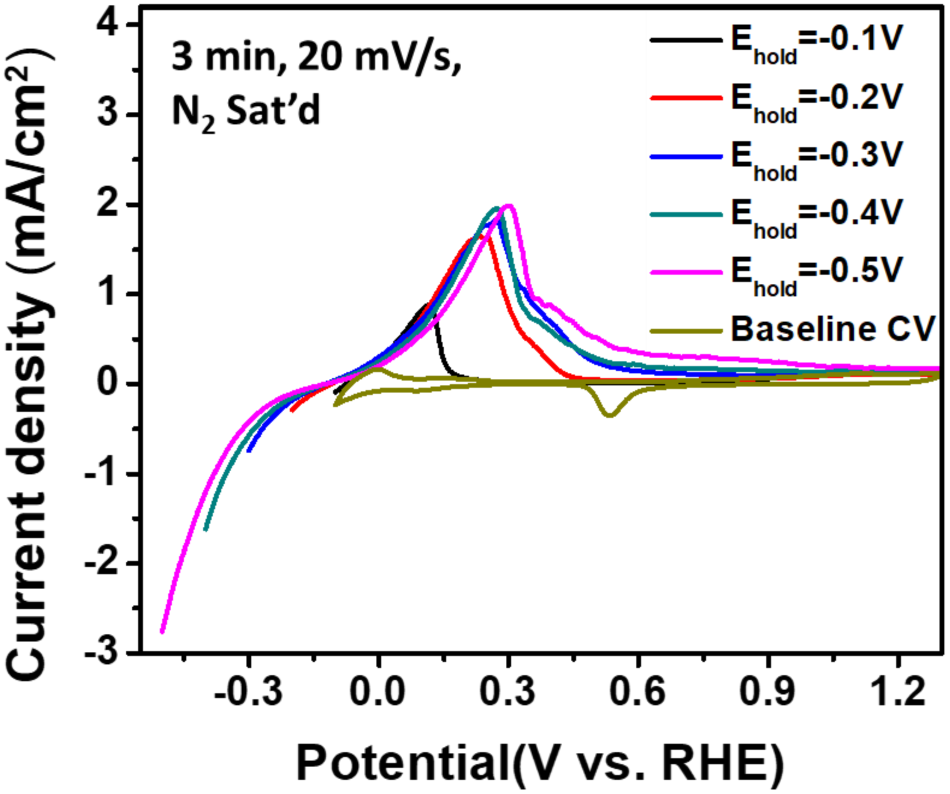

Supplementary Fig. 16 Positive LSV and baseline CV in N_2 sat 0.1M $KHCO_3$ electrolyte after holding the potential at various applied overpotentials (-0.1 to -0.5 V vs RHE) for 3 mins.

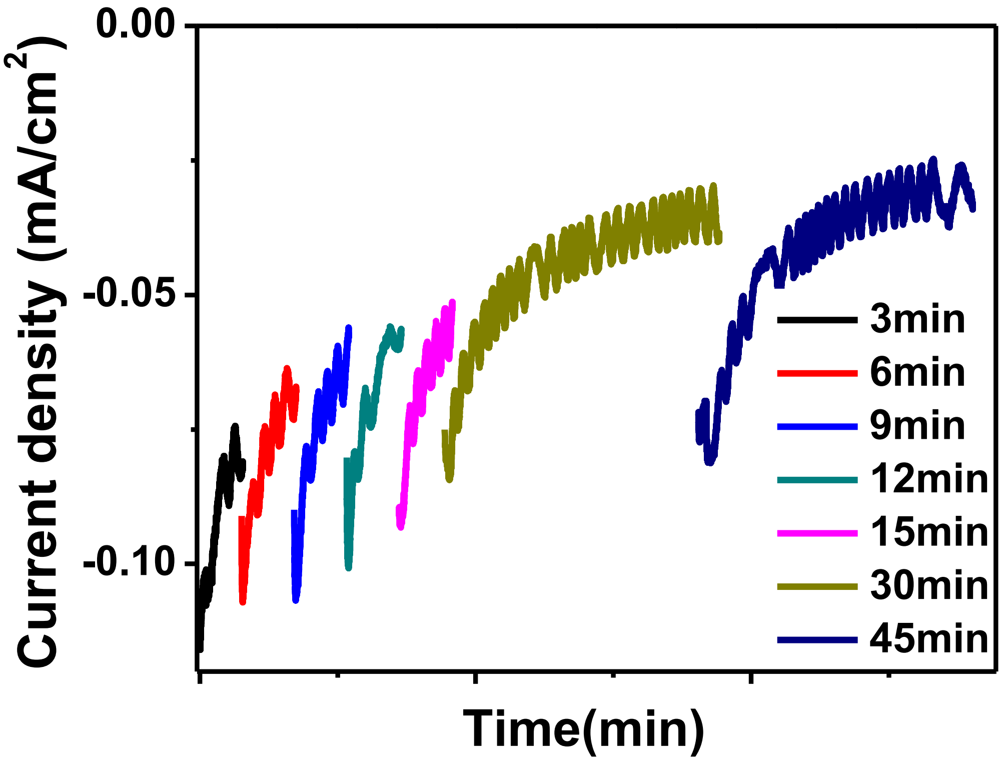

Supplementary Fig. 17 Current density as a function of time of the large-format Pd-decorated glassy carbon electrode, which holds at potential of -0.1 V vs. RHE at various durations in CO_2-saturated 0.1 M $KHCO_3$ solution, demonstrating the effect of *CO poisoning on the current density. The electrochemical surface area (ECSA) of the Pd/PdH_x particles was determined using double-layer capacitance measurements before ($ECSA_{t=0}$) and after each electrolysis time ranging from 3 to 45 minutes ($ECSA_{t=3\ to\ t=45}$)

Supplementary Note 5:

ECSA Calculations

Linear fitting of the maximum current at different scan rates of 20 to 120 mV s^{-1} was necessary for the accurate determination of the Cdl at the solid/liquid interface to measure the electrochemical active surface area (ECSA) that was calculated by the following Eq:

$$\mathbf{ECSA} = \frac{\mathbf{C_{dl}}}{\mathbf{C_s}}$$

Where C_s is the standard specific capacitance, that is 0.027 mF/ cm^2 for a flat glassy carbon electrode.

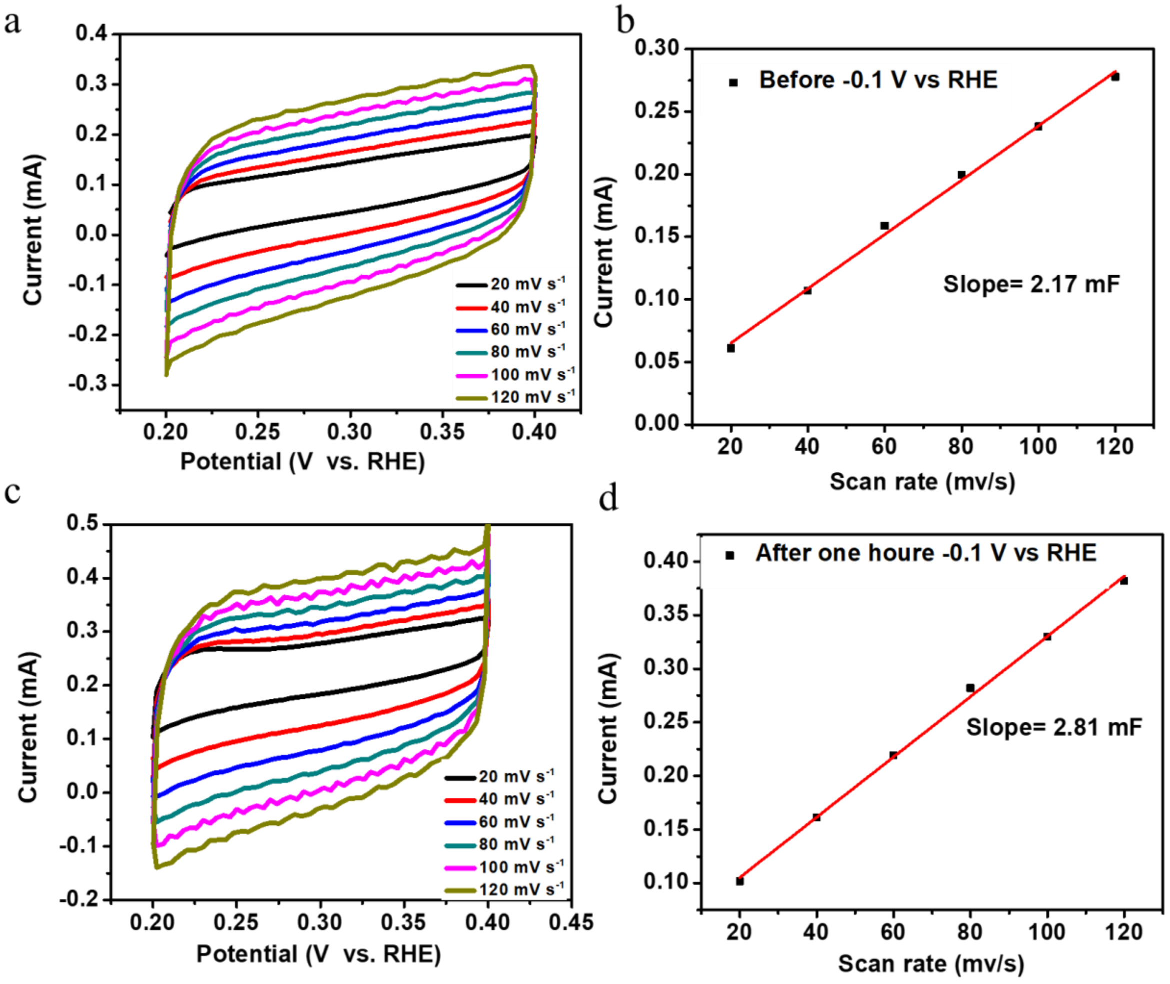

Supplementary Fig. 18 CV scans at different scan rates from 20 to 100 mV s^{-1} of Pd particles in 0.1M $KHCO_3$, CO_2 saturated electrolyte: **a-b** before and **c-d** after electrolysis under an applied potential of -0.1 V vs RHE.

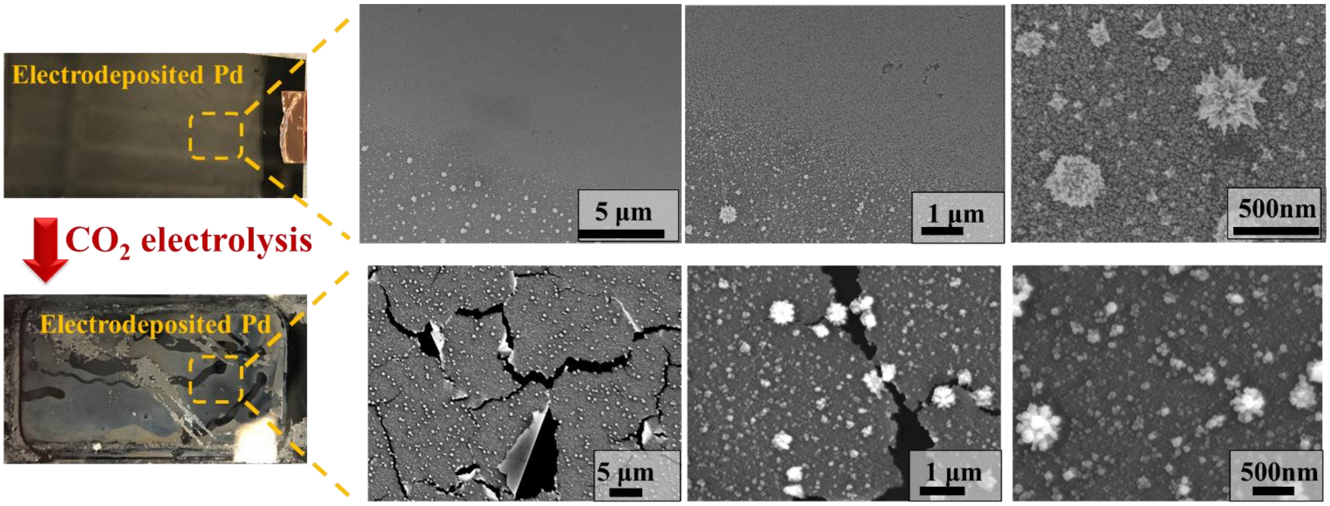

Supplementary Fig. 19 Optical images and SEM before and after one hour of electrolysis at an applied potential of -0.5 V vs RHE in CO_2 saturated 0.1 M $KHCO_3$ for the palladium-decorated large-format glassy carbon electrode.

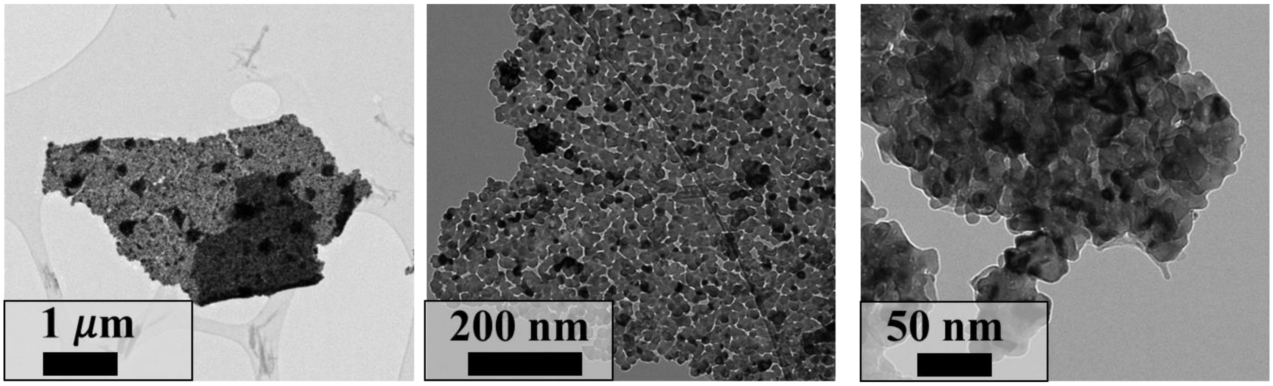

Supplementary Fig. 20 TEM images of the palladium decorated large-format glassy carbon electrode after one hour of electrolysis at -0.5 V vs RHE in CO_2 saturated 0.1 M $KHCO_3$.

Supplementary note 6:

DFT calculations:

The reported DFT-based (constant potential) energies were calculated using the constant-potential mode of SJM[4] implemented in GPAW [5,6].

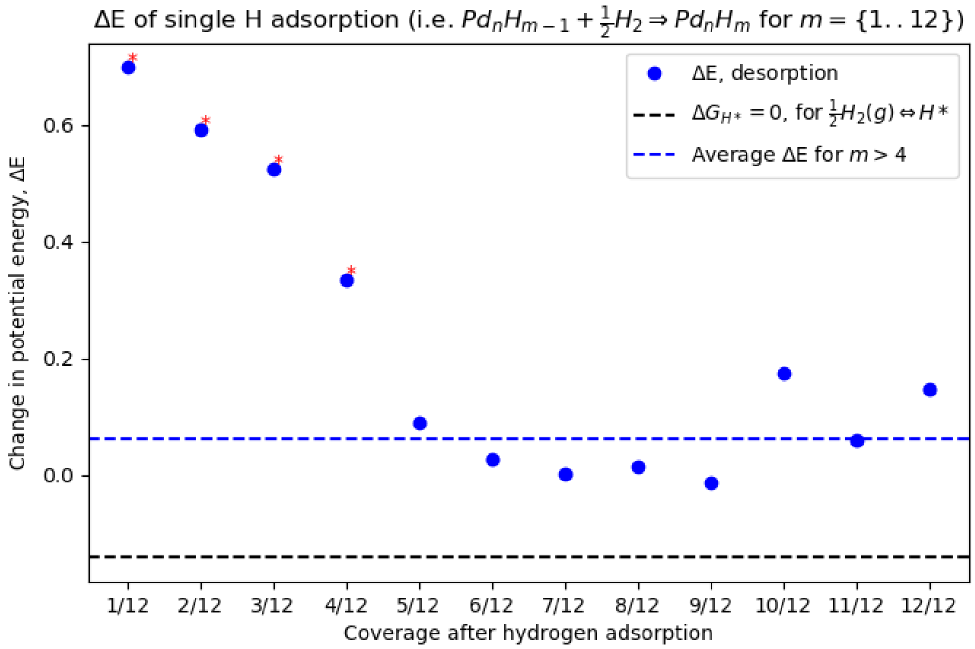

Supplementary Fig. 21 Differential adsorption energies for hydrogen adsorption into hollow sites of a beta palladium hydride surface. In the structures marked with an asterisk, there is hydrogen migration from the subsurface to the surface, which convolutes the adsorption energy picture. For the remaining 8 coverages, the average change in potential energy (blue line) is roughly 0.05 eV. Accounting for the entropy loss (black line), the hydrogen adsorption is roughly 0.2 eV uphill in energy at 0 V vs RHE, meaning it will be thermoneutral around -0.2 V vs RHE.

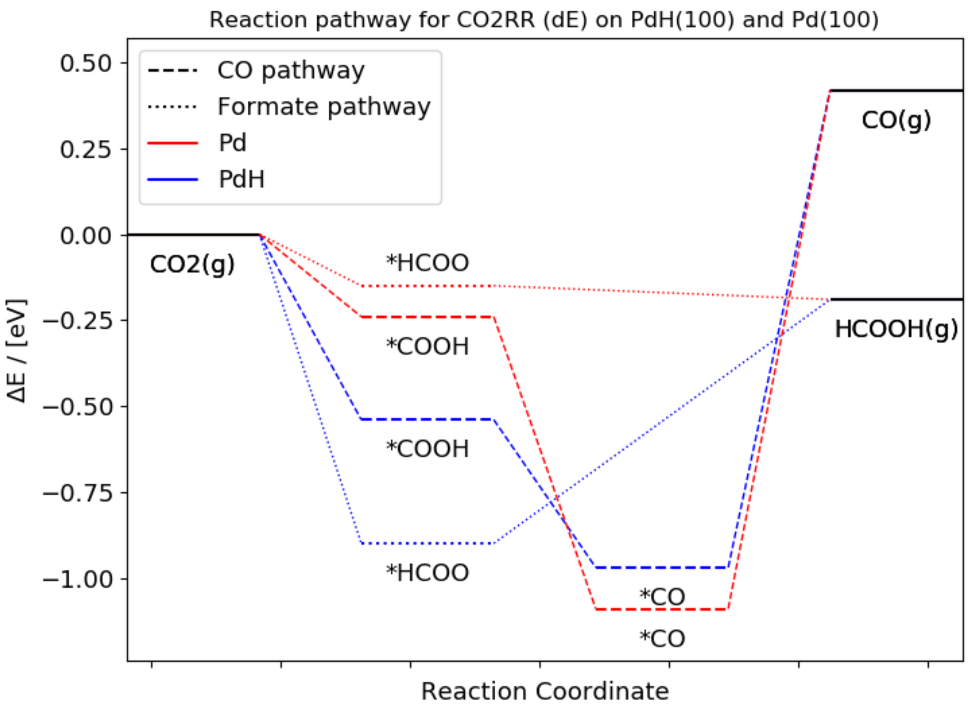

Supplementary Fig. 22 Potential energy diagram showing suggested reaction pathways for CO_2 towards either CO or formic acid on β-PdH_x(100) (blue) and Pd(100) (red). The dotted lines show the formate/formic acid pathway while the striped lines show the CO pathway. On both surfaces, *CO is the most stable intermediate and as *CO is expected to show the same response to potential as the two possible products, the (100) facet is expected to be poisoned by CO under reaction conditions.

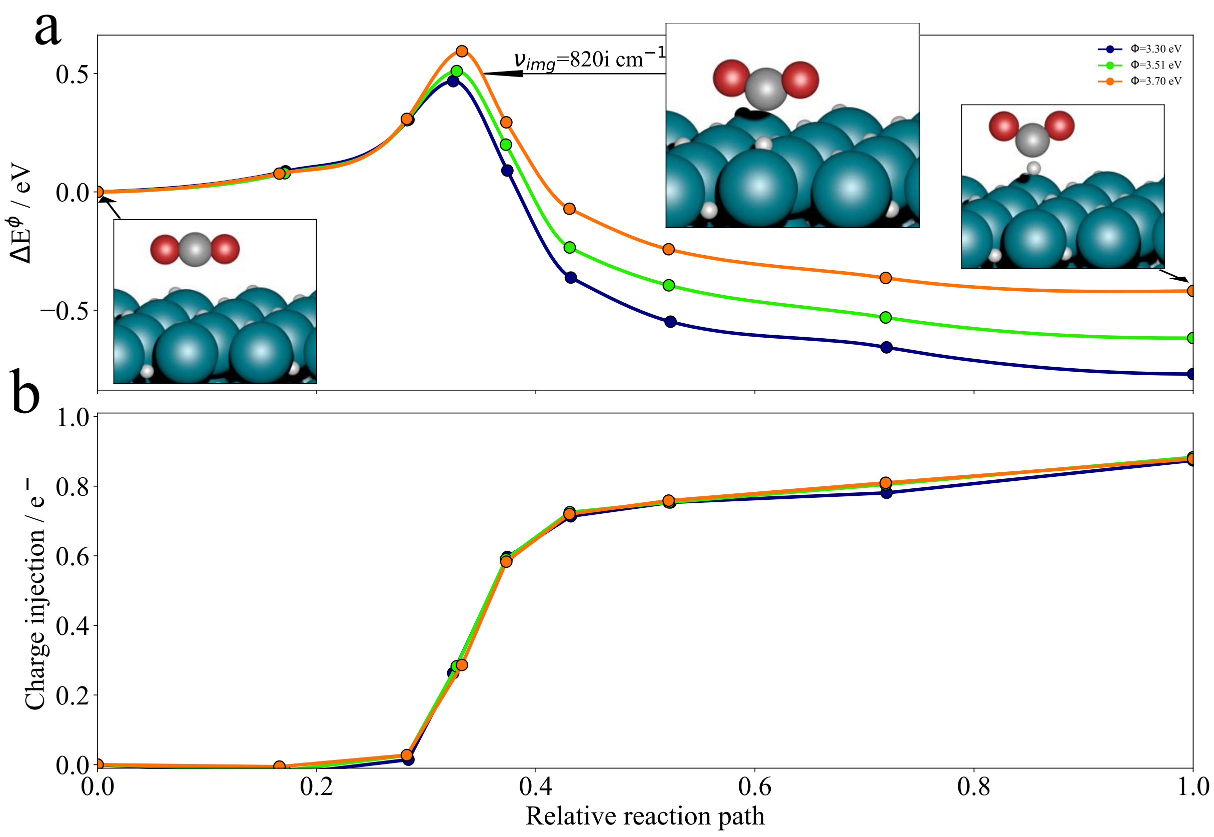

Supplementary Fig. 23 a Calculated minimum energy pathways of the CO_2 hydrogenation step towards formate. **b** The accompanied electron injection into the unit cell in the course of the reaction in order to keep the potential constant. Three different potentials are shown with varying colors, where the work functions given in the legend can be related to the SHE potential by subtracting the value of 4.4eV from them[7]. Note that the charge injection at the transition state is about 0.25 at all potentials, this leads to a fairly small potential response. Further note that in the Free energy diagram shown in the main text, the initial and final states do not correspond to the gas phase molecules hovering above the slab, but rather consist of separate calculations of the gas species and the PdH_x slabs.

References:

1 Wang, X. et al. Morphology and mechanism of highly selective Cu (II) oxide nanosheet catalysts for carbon dioxide electroreduction. Nature communications **12**, 1-12 (2021).

2 Egerton, R. F. Electron energy-loss spectroscopy in the electron microscope. (Springer Science & Business Media, 2011).

3 Holtz, M. E., Yu, Y., Gao, J., Abruña, H. D. & Muller, D. A. In situ electron energy-loss spectroscopy in liquids. Microscopy and Microanalysis **19**, 1027-1035 (2013).

4 Kastlunger, G., Lindgren, P. & Peterson, A. A. Controlled-potential simulation of elementary electrochemical reactions: proton discharge on metal surfaces. The Journal of Physical Chemistry C **122**, 12771-12781 (2018).

5 Mortensen, J. J., Hansen, L. B. & Jacobsen, K. W. Real-space grid implementation of the projector augmented wave method. Physical Review B **71**, 035109 (2005).

6 Enkovaara, J. et al. Electronic structure calculations with GPAW: a real-space implementation of the projector augmented-wave method. J Phys Condens Matter **22**, 253202 (2010). https://doi.org:10.1088/0953-8984/22/25/253202

7 Trasatti, S. The absolute electrode potential: an explanatory note (Recommendations 1986). Pure and Applied Chemistry **58**, 955-966 (1986).
