# Supplementary material for: Impact of palladium/palladium hydride conversion on electrochemical CO2 reduction via in-situ transmission electron microscopy and diffraction
Source: Nat Commun. 2024 Jan 31;15:938. doi: 10.1038/s41467-024-45096-3 (PMC10831057; doi:10.1038/s41467-024-45096-3)
Supplement: Supplementary file 3 — Description of Additional Supplementary Files [file 41467_2024_45096_MOESM3_ESM.pdf]

Description of Additional Supplementary Files

Supplementary Movie 1: Showing in-situ LP-HAADF-STEM of Pd particles held at an electrode potential switching between 1.2 V and -0.2 V vs RHE in CO2 saturated 0.1M KHCO3, demonstrating the enlargement of the Pd particles as a function of potential. Images were collected with an electrolyte flow rate of 5 µL/min and a beam dose of 39.7 e-/nm2.sec, acquisition frame rate of 1 frame per second, and a video playback rate of 5 frames per second.

Supplementary Movie 2: Showing in-situ LP-TEM/SAD patterns collected at electrode potentials switching between 1.2 V and -0.2 V vs RHE in CO2 saturated 0.1M KHCO3. A contraction of the SAD diffraction rings (due to lattice expansion) upon applying an electrode potential of -0.2 V vs RHE are observed. Diffraction patterns were collected with an electrolyte flow rate of 5 µL/min and a beam dose of 39.7 e-/nm2.sec, an acquisition frame rate of 1 frame per second, and a video playback rate of 1 frame per second. The observed lattice expansion is attributed to hydrogen absorption and intercalation into Pd, resulting in the formation of different PdHx phases.

Supplementary Movie 3 : Showing in-situ LP-HAADF-STEM of Pd/PdHx particle migration, agglomeration, and detachment from the glassy carbon working electrode at an applied potential of -0.2 V vs. RHE in CO2 saturated 0.1M KHCO3. Images were collected with an electrolyte flow rate of 5 µL/min and a beam dose of 39.7 e-/nm2.sec, an acquisition frame rate of 1 frame per second and a video playback rate of 1 frames per second.

Supplementary Movie 4 : Showing in-situ LP-HAADF-STEM of Pd/PdHx particle detachment from the glassy carbon working electrode at an applied potential of -0.2 V vs. RHE in CO2 saturated 0.1M KHCO3. Images were collected with an electrolyte flow rate of 5 µL/min and a beam dose of 39.7 e-/nm^2.sec, an acquisition frame rate of 1 frame per second and a video playback rate of 10 frames per second.
